# Application of oXiris-continuous hemofiltration adsorption in patients with sepsis and septic shock: A single-centre experience in China

**DOI:** 10.3389/fpubh.2022.1012998

**Published:** 2022-09-29

**Authors:** Yanyan Zhou, Chenfang Wu, Lin Ouyang, Ying Peng, Dingming Zhong, Xiaohong Xiang, Jinxiu Li

**Affiliations:** Department of Critical Care Medicine, The Second Xiangya Hospital of Central South University, Changsha, China

**Keywords:** oXiris, continuous hemofiltration adsorption, sepsis, septic shock, cytokine storm

## Abstract

oXiris is a new, high-adsorption membrane filter in continuous hemofiltration adsorption to reduce the inflammatory response in sepsis. The investigators retrospectively reviewed patients with sepsis/septic shock who underwent at least one oXiris-treatment from November 2020 to March 2022. The demographic data, baseline levels before treatment, clinical datas, prognosis, and the occurrence of adverse events during treatment were recorded. 90 patients were enrolled in this study. The hemodynamic indices, sequential organ failure assessment score, lactate, inflammatory biomarkers levels were significantly improved at 12 h and 24 h after treatment. Procalcitonin and interleukin-6 reduction post-treatment of oXiris were most pronounced in infection from skin and soft tissue, urinary and abdominal cavity. Logistic regression analysis showed that pre-treatment sequential organ failure assessment score (*p* = 0.034), percentage decrease in sequential organ failure assessment score (*p* = 0.004), and age (*p* = 0.011) were independent risk factors for intensive care unit mortality. In conclusion, oXiris-continuous hemofiltration adsorption may improve hemodynamic indicators, reduce the use of vasoactive drugs, reduce lactate level and infection indicators. Of note, oXiris improve organ function in sepsis, which may result to higher survival rate.

## Introduction

Sepsis is a life-threatening organ dysfunction syndrome because of a disordered host response after a host infection caused by pathogenic microorganisms (1). Sepsis has been confirmed to be associated with a >10% in-hospital mortality; septic shock is subset of sepsis with particularly severe circulatory, cellular, and metabolic abnormalities that carries a greater risk of death than sepsis alone, and it is associated with >40% in-hospital mortality (1). Although the concept and technology of treatment have developed rapidly, the incidence and case fatality rate of sepsis are still high, which has become challenge for the global medical community.

Sepsis is a highly heterogeneous clinical syndrome. Different host response and pathophysiological driving mechanisms of different patients result in complex but excessive immune activation and immunosuppression of sepsis, which has always been the central link in the pathophysiology of sepsis (2). Upregulation of pro-inflammatory and anti-inflammatory pathways causes a system-wide release of cytokines, mediators, and pathogenesis-related molecules, resulting in the activation of the coagulation and complement cascades (3). In septic shock, the dysregulated host response to infectious pathogens leads to a cytokine storm—the uncontrolled production and release of humoral pro- and anti-inflammatory mediators—causing cytotoxicity and promoting the development of organ dysfunction and increased mortality (4).

Continuous renal replacement therapy (CRRT) is the main form of RRT in the intensive care unit (ICU), because it has accurate volume control and stable acid-base and electrolyte correction and can achieve hemodynamic stability. Significant results have been achieved in severe patients, especially in the field of sepsis treatment (4, 5). In addition to renal replacement therapy, adsorption therapy seems to be more promising in the application of sepsis (3, 5, 6).

oXiris (Baxter, Meyzieu, France) is a filter that combines cytokine- and endotoxin-removal properties, renal replacement function, and antithrombotic properties (7). The peak of “cytokine casade” should be within the first a few hours of onset, and this should be the optimal intervening window for oXiris. It can reduce the levels and early harmful effects of circulating proinflammatory cytokines and endotoxins in the first few hours and days of septic shock therapy to improve patient outcomes (4, 7).

It has been shown that oXiris treatment in septic patients enables optimization of hemodynamic status, clears inflammatory mediators such as tumor necrosis factor (TNF)-α, interleukin (IL)-6, IL-8, and interferon-γ, and ultimately improves prognosis (8–11). However, the evidence-based use of oXiris for sepsis is still limited. This study aims to explore the clinical effect of continuous hemofiltration adsorption (CHFA) with oXiris filter in patients with sepsis/septic shock.

## Materials and methods

### Study population

The study protocol was approved by the Institutional Ethics Committee of the Second Xiangya Hospital of Central South University (No. 2022K040). We retrospectively collected data on 90 patients with sepsis/septic shock who received at least one oXiris-CHFA treatment at the Second Xiangya Hospital, Central South University, between November 2020 and March 2022. All the patients treated in ICU. The inclusion criteria were: (1) Patients with clinical diagnosis consistent with sepsis (meeting the 2016 Sepsis-3 definition) (1); and (2) men or women in the age range of 18–90 years. The exclusion criteria were: (1) immunodeficiency diseases such as tumors, connective tissue disease, and use of immunosuppressants in the last 3 months; and (2) pregnant or lactating patients.

### Methods

All selected patients underwent titrated fluid resuscitation in strict accordance with the sepsis guidelines (2016 Sepsis-3) (1) and received vasoactive drugs, empiric/based antibiotics, mechanical ventilation, sedation, and analgesia. Baseline characteristics, primary site of infection, microbiological results, antibiotic and acute physiological and chronic health assessment (APACHE II) scores, initial creatinine level, and renal function grade [acute kidney injury (AKI)- stages 1–3 (12) or end-stage renal disease (ESRD)] were recorded. Details of baseline characteristics and infection and clinical outcomes are presented in Table 1.

**Table 1:** Baseline characteristics and infection and clinical outcomes of included patients.

**Clinical data**	
Male (*n*, %)	59 (65.6)
Age, years	63 [50.75–74]
SOFA total	14 [10-17]
APACHE II	24 [19-32]
Creatinine, μmol/L	172.00 [109.75–278.50]
**Renal function at CRRT initiation (** ***n*** **, %)**	
AKI Stage 3	24 (26.7)
AKI Stage 2	23 (25.6)
AKI Stage 1	32 (35.6)
ESRD	11 (12.2)
Norepinephrine (*n*, %)	84 (87.00)
Norepinephrine, μg/kg/min	0.6 [0.14–1.50]
Mechanical ventilation (*n*, %)	75 (83.3)
PaO_2_/FiO_2_ ratio	190.25 [112.85–287.00]
ECMO (*n*, %)	5 (5.5)
Lactate ≥2 mmol/L (*n*, %)	77 (85.56)
Lactate, mmol/L	5.15 [2.78–8.65]
**Infection characteristics**	
**Site of infection (n, %)**	
Pulmonary	28 (31.1)
Abdominal	33 (36.7)
Skin and soft tissue	5 (5.6)
Bacteremia	16 (17.8)
Urinary	8 (8.9)
**Culture (** ***n*** **, %)**	
Gram negative	45 (50.0)
Gram positive	17 (18.9)
Fungus	12 (13.3)
Not identified	16 (17.8)
Positive blood culture	27 (30)

SOFA, Sequential Organ Failure Assessment; APACHE II, Acute Physiology and Chronic Health Evaluation II; AKI, acute kidney injury; CRRT, continuous renal replacement therapy; ESRD, end-stage renal disease; ECMO, extracorporeal membrane oxygenation. Continuous variables are presented as median [interquartile range].

All patients received at least one oXiris-CHFA treatment with the oXiris filter on a Prismaflex system (Baxter International, Deerfield, IL, United States). The mode was CHFA (CVVH/CVVHDF + oXiris adsorption), and 4 patients underwent hemoperfusion therapy (HA380, Jafron Biomedical Co., Zhuhai, China). Start timing, treatment dose, duration, and anticoagulation (citrate/heparin/no anticoagulant) were determined by the physician in charge according to the patient's specific situation. The oXiris-CHFA treatment duration lasted at least 24 h for each patient, except under special circumstances (such as death or abandoning treatment). The blood flow rate was maintained between 150 and 200 mL/min. Details of the CRRT prescriptions are presented in Table 2.

**Table 2:** CRRT prescription in each patient.

**CRRT parameter**	
**CRRT modality (** ***n*** **, %)**	
CVVH	56 (62.2)
CVVH + HP	4 (4)
CVVHDF	30 (33)
**Blood flow rate (** ***n*** **, %)**	
150 mL/min	39 (43.3)
200 mL/min	51 (56.7)
**Circuit anticoagulation (** ***n*** **, %)**	
Citrate	39 (43.3)
Heparin	5 (5.6)
None	46 (51.1)
Prescribed therapeutic dose (mL/kg/h) (*n*, %)	28.95 [26.81–38.89]
≥30 mL/kg/h	38 (42.2)
<30 mL/kg/h	52 (57.8)
Filtration fraction (%)	21.15 [19.03–24.31]
Time between ICU admission and oXiris^®^ initiation, h	18.00 [7.00–61.50]
Number of sessions per patient	2.00 [1.00–2.00]
Duration of CRRT treatment, h	66.85 [37.63–132.50]
Duration of oXiris-CHFA treatment, h	38.5 [22.00–59.87]

CRRT, continuous renal replacement therapy; CVVH, continuous venovenous hemofiltration; HP, hemoperfusion; CVVHDF, continuous venovenous hemodiafiltration; ICU, intensive care unit. Continuous variables are presented as median [interquartile range].

### Data collection

For patients who had used oXiris-CHFA several times, we only recorded data before and after the first treatment, including heart rate (HR), respiratory rate (RR), mean arterial pressure (MAP), and norepinephrine (NE) level at 0, 12, and 24 h after treatment. The levels of procalcitonin (PCT), IL-6, and lactate and the sequential organ failure assessment (SOFA) scores were compared at 0, 12, and 24 h after treatment.

### Study design and statistical methods

This study was designed for self-pairing, and SPSS software (version 22.0; IBM Corporation, Armonk, NY, United States) was used for statistical analysis of data. The distribution of measurement data was first tested, and normally distributed measurement data were expressed as mean ± standard deviation, and repeated measurement ANOVA test was used for comparison among three groups. Non-normally distributed measurement data were expressed as median [interquartile range (IQR)]: Friedman's test was used for comparison among three groups, and Wilcoxon symbol rank test was used for comparison between two groups. A *p* value < 0.05 was considered to indicate statistically significant differences. Based on patients' survival during ICU treatment, all patients were divided into survival and non-survival groups. Two independent non-normally distributed samples were compared by Mann–Whitney *U* test, and categorical variables were compared using either chi-square test or Fisher's exact test, as appropriate. Independent sample Kruskal-Wallis test was used to compare subgroups grouped according to baseline conditions and RRT parameters.

## Results

### Demographic data

A total of 90 patients (59 male; median age: 63 years; IQR: 50.75–74 years) with sepsis/septic shock were included from November 2020 to March 2022. Eleven patients had ESRD but required CRRT due to hemodynamic instability. Of the 79 patients with AKI, 32, 23, and 24 patients had stages 1, 2, and 3, respectively. On admission, the median APACHE II score was 24 (IQR: 19–23). The median dose of initial use of the vasoactive drug was 0.6 μg/kg/min (IQR: 0.14–1.50). The median oxygenation index was 190.25 mmHg (IQR: 112.85–287.00): 75 patients were undergoing mechanical ventilation for respiratory support at inclusion, and 5 patients were treated with extracorporeal membrane oxygenation (ECMO). The median lactate level was 5.15 mmol/L (IQR: 2.78–8.65), and 77 patients had lactate levels ≥2 mmol/L. Abdominal infection (*n* = 33) was the most common source of sepsis in this study, followed by pulmonary infection (*n* = 28). Gram-negative sepsis was found in 45 (50%) patients, followed by gram-positive (*n* = 17) and fungal (*n* = 12) sepsis. Further, 27 patients (30%) had positive blood cultures. Patient characteristics and the details of the infections are described in Table 1.

All patients were treated with 1–12 oXiris-CHFA [median = 2 (IQR: 1–2)] at the discretion of the attending physician. The median time between ICU admission and the start of oXiris was 18 h (IQR: 7.00–61.50), and in terms of treatment mode selection, 56 patients required CVVH, 30 required CVVHDF, and 4 required CVVH + HP. More than half of the patients (51.10%) required no anticoagulant, 43.30% had citrate anticoagulant, and only 5.60% had heparin anticoagulant therapy. The median prescribed treatment dose was 28.95 mL/kg/h (IQR: 26.81–38.89), and the median filter score was 21.15% (IQR: 19.03%−24.31%). Because some patients had also been treated with other filters, the median CRRT treatment time for each patient was 66.85 h (IQR: 37.63–132.50), and the oXiris treatment time was 38.5 h (IQR: 22.00–59.87). Details of the CRRT prescriptions are presented in Table 2.

### Results after oXiris treatment

**Figure 1:**
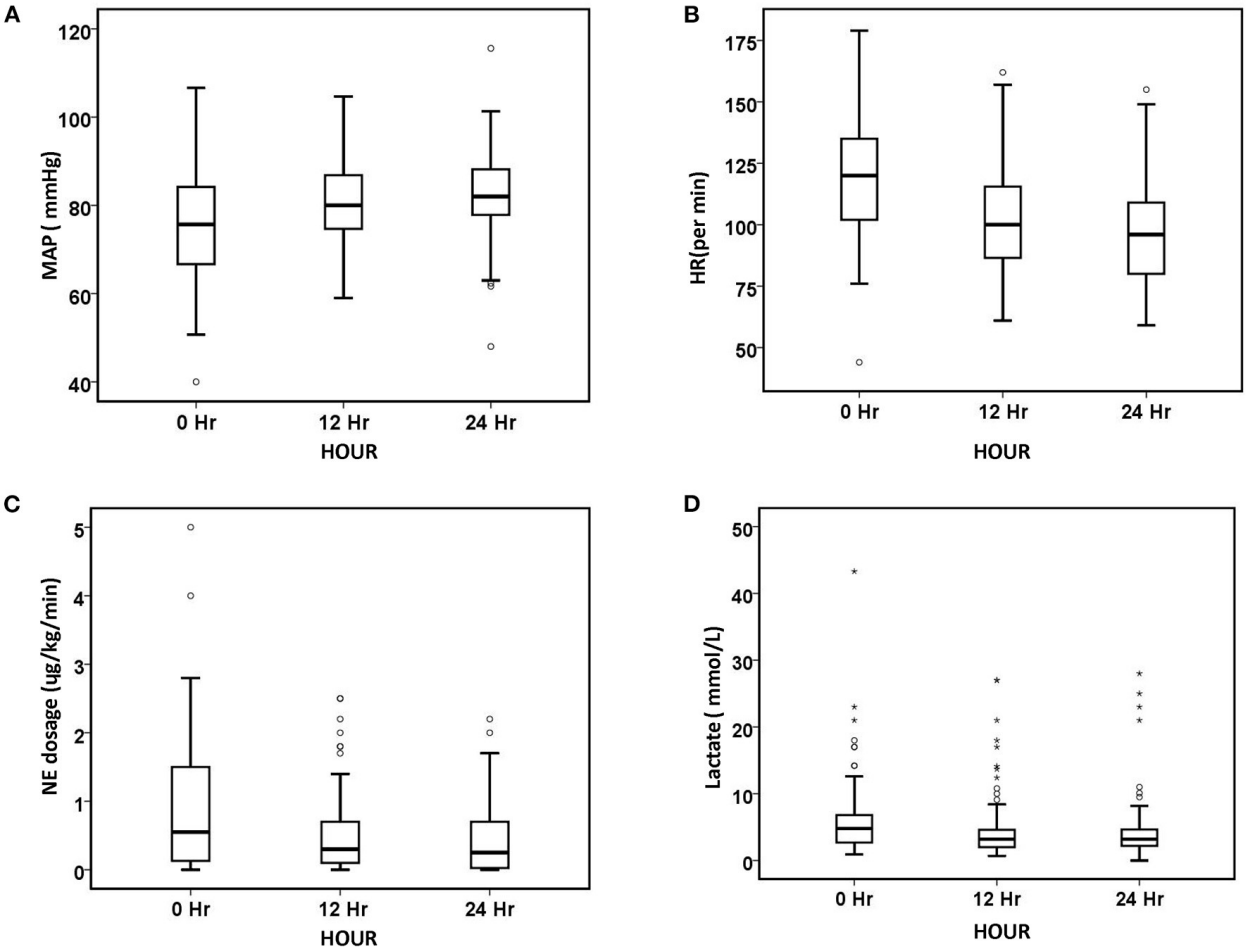
Changes in hemodynamic parameters and lactate level over 24 h. **(A)** MAP, **(B)** HR, **(C)** NE dosage, and **(D)** lactate. The number of patients at baseline, 12 h, and 24 h was 90, 85, and 75, respectively. The symbol * stands for outlier.

**Figure 2:**
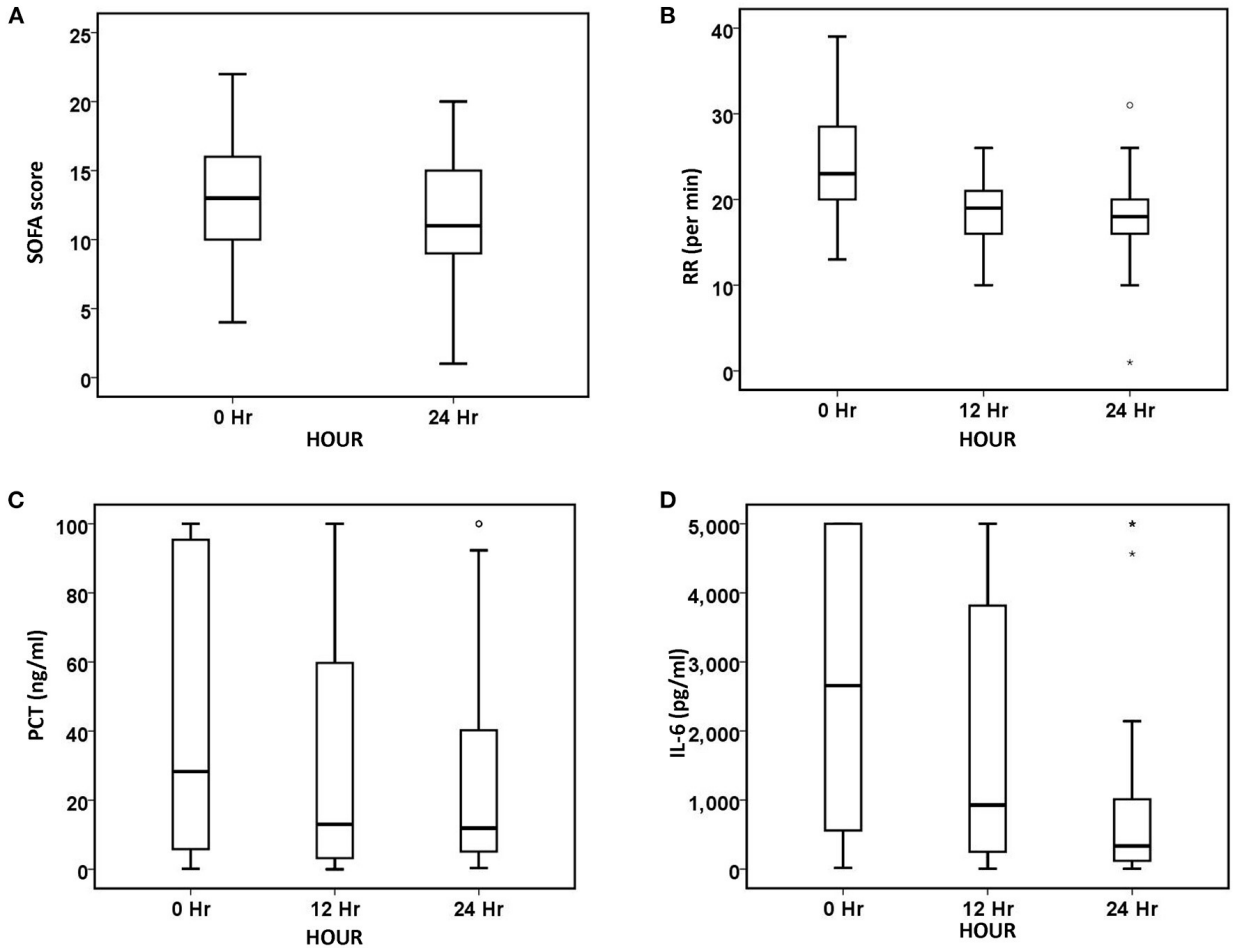
Changes in organ function and inflammatory biomarkers over 24 h. **(A)** SOFA, **(B)** RR, **(C)** PCT, and **(D)** IL-6. The number of patients at baseline, 12 h, and 24 h was 90, 85, and 75, respectively. The symbol * stands for outlier.

After 24 h of oXiris-CHFA, MAP increased by 9.1% (*p* < 0.001), NE dose decreased by 61.53% (*p* < 0.001), HR decreased by 21.31% (*p* < 0.001) and RR decreased by 21.74% (*p* < 0.001). In parallel to hemodynamic stabilization, blood lactate levels decreased by 37.86% after 24 h compared to the pre-treatment period (*p* = 0.008) (Table 3, Figure 1). The SOFA score was significantly decreased by 21.43% (*p* < 0.001) after 24 h of oXiris-CHFA treatment, and the median pre-/post-treatment SOFA score was 14 (10.00–17.00) vs. 11.00 (9.00–15) (Table 3, Figure 2).

**Table 3:** Hemodynamic and inflammatory biomarkers, metabolic changes, and blood platelet count during oXiris-CHFA treatment.

**Parameter**	**Baseline (*n* = 90)**	**12 h (*n* = 85)**	**24 h (*n* = 75)**	***p* value**
MAP^a^, mmHg	75.16 [66.00–84.00]	79.33 [73.00–85.84]	82.00 [77.67–88.67]	< 0.001
Norepinephrine dosage^a^, μg/kg/min	0.65 [0.14–1.50]	0.40 [0.12–1.00]	0.25 [0.00–0.70]	<0.001
HR^a^, per min	122.00 [102.75–136.50]	101.00 [87.50–119.00]	96.00 [80.00–109.00]	<0.001
RR^a^, per min	23.00 [19.75–29.00]	20.00 [16.50–21.00]	18.00 [16.00–20.00]	<0.001
SOFA^b^	14 [10.00–17.00]	–	11.00 [9.00–15]	<0.001
Lactate^a^, mmol/L	5.15 [2.78–8.65]	3.6 [2.05–7.40]	3.20 [2.20–4.80]	0.008
Procalcitonin^a^, ng/mL	23.70 [3.31–81.07]	12.9 [2.47–59.75]	11.90 [5.08–41.90]	<0.001
Interleukin-6^a^, pg/mL	1986.50 [555.25–5000.00]	1245.00 [292.00–5000.00]	361.50 [138.75–1051.00]	<0.001
Blood platelet count^a^, 10^9^/L	83.50 [28.75–143.50]	–	44.00 [22.00–90.00]	<0.001

oXiris-CHFA, continuous hemofiltration adsorption with oXiris; MAP, mean arterial pressure; HR, heart rate; RR, respiratory rate; SOFA, Sequential Organ Failure Assessment. Non-normally distributed measurement data were expressed as median [interquartile range].

^a^Friedman's test for comparison between three groups.

^b^Wilcoxon symbol rank test for comparison between two groups.

p value < 0.05 was considered as a significant difference.

In terms of infection index, PCT decreased by 48.79% (*p* < 0.001) after 24 h of treatment, and IL-6 decreased by 81.80% (*p* < 0.001) (Table 3, Figure 2). The ICU mortality rate was 34.4%, wherein 59 patients survived and 31 patients died. The median ICU stay time was 7.5 days (4.00–20.50), and the 30-day mortality rate was 44.4%. Hospital mortality rate was 35.6%.

### Subgroup analysis and regression analysis

#### Initiation time and therapeutic dose of oXiris-CHFA

**Figure 3:**
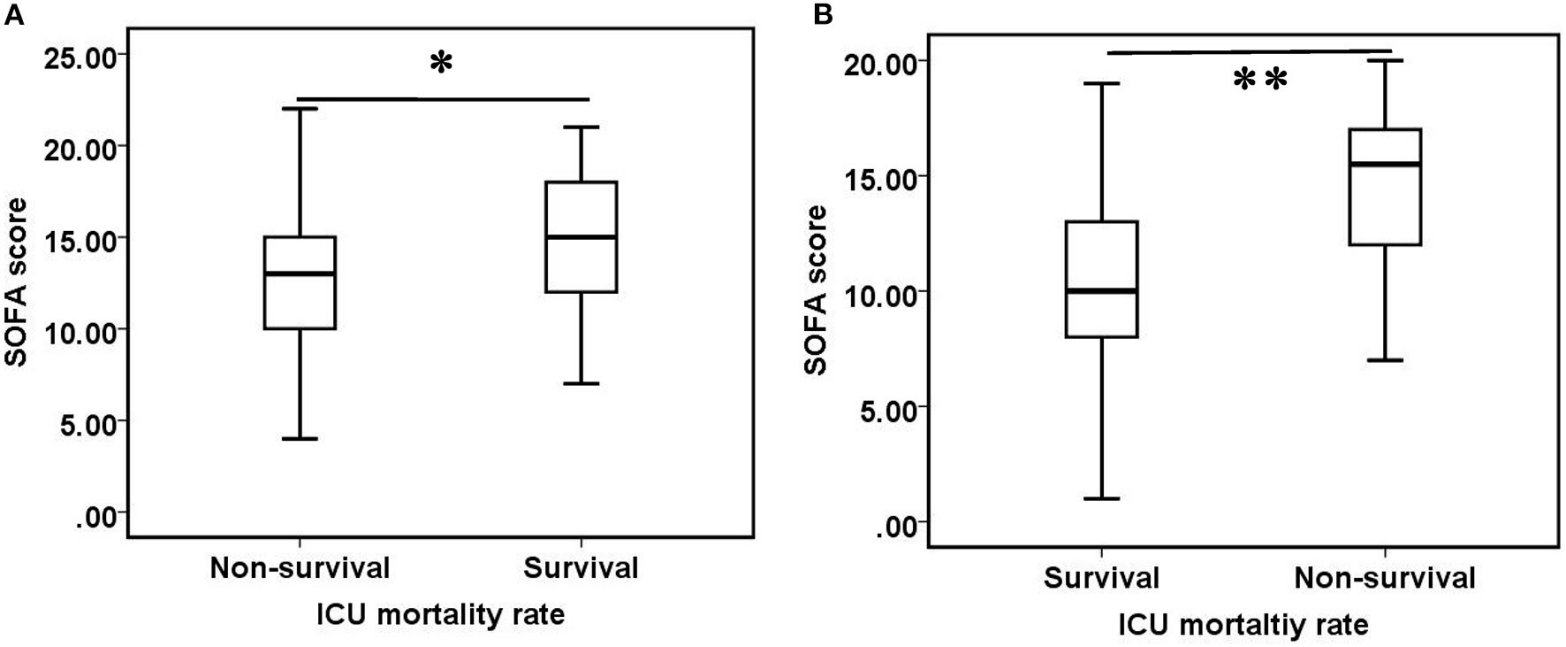
Comparison of SOFA scores between survival and non-survival groups. **(A)** Before oXiris-CHFA treatment (0 h), **(B)** after oXiris-CHFA treatment (24 h). *P = 0.064, **P < 0.001.

There were no statistically significant differences in ICU mortality rate between patients who received oXiris ≤ 24 h or >24 h (*p* = 0.921) and between patients whose prescription therapeutic dose was ≥30 mL/kg/h or < 30 mL/kg/h (*p* = 0.309). There was no significant difference in SOFA scores between surviving and non-surviving groups [13.00 (10.00–16.00) vs. 14.00 (12.00–18.00), *p* = 0.064], but after 24 h of oXiris-CHFA, the surviving patients had significantly lower SOFA scores than non-surviving patients [10.00 (8.00–13.00) vs. 15.50 (12.00–17.00), *p* < 0.001] (Figure 3).

#### Site of primary infection

**Figure 4:**
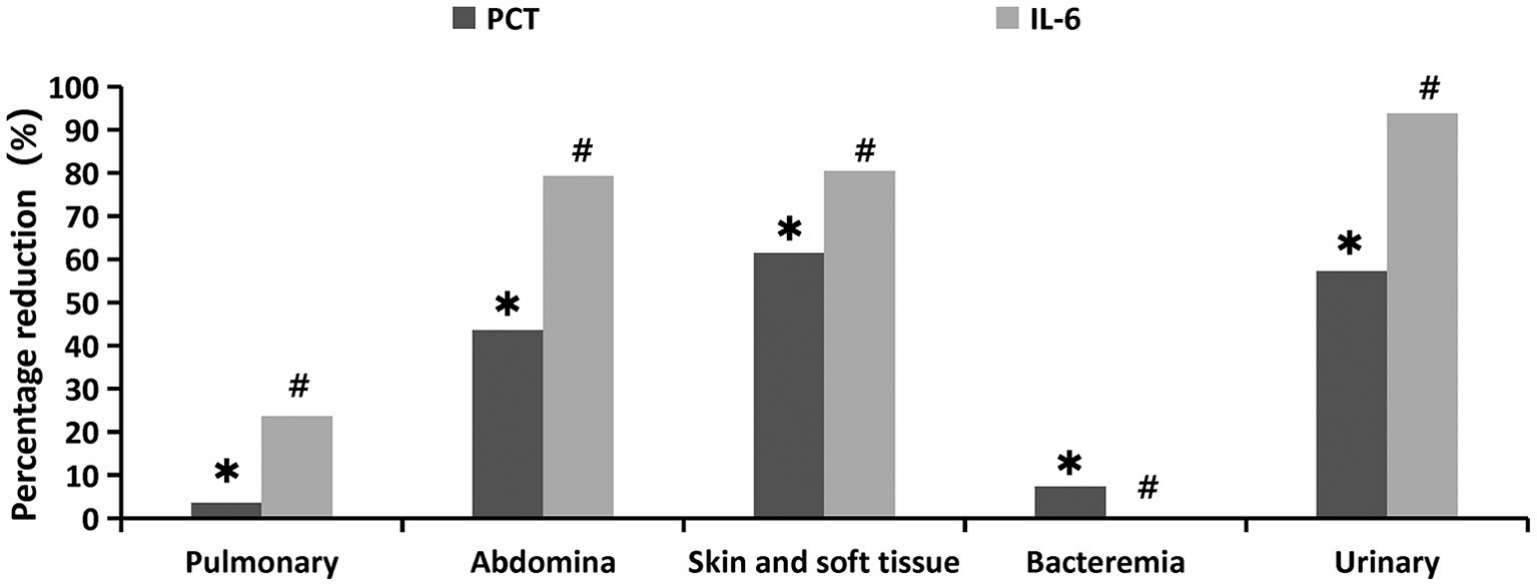
Percentage reduction of infection indicators at different primary infection sites 24 h after oXiris-CHFA treatment. *P = 0.035, ^**#**^*p* = 0.001.

From the perspective of primary infection sites, the distribution of PCT and IL-6 percentage reduction was significantly (*p* = 0.035 and *p* = 0.001, respectively). The decline in PCT was most pronounced in skin and soft tissue infections (61.4%), followed by urinary (57.20%) and abdominal (43.63%) infections, and was least in blood (7.35%) and pulmonary infections (3.57%). The decrease in IL-6 was also the most pronounced in urinary tract infections (93.8%), followed by skin and soft tissue infection (80.42%), abdominal cavity infection (79.29%), and pulmonary infection (23.64%), and showed the least significant decrease in blood infection (0%) (Figure 4, Supplementary Table 1).

#### Other subgroup analyses

We divided the sample population into groups from different perspectives, such as renal function status at CRRT initiation, culture result type, CRRT method, blood flow velocity, and anticoagulation method, and compared whether there were differences in PCT, IL-6, SOFA score, the decline of lactate, vasoactive drug dosage, and the improvement of vital signs among the groups. The results showed that patients who started treatment at AKI1 stage had a greater decrease in IL-6 levels than those who started treatment later or had end-stage renal disease (*P* = 0.027) (Supplementary Table 1). Patients with a blood flow rate of 200 ml/min during CRRT showed more significant improvements in HR (*p* = 0.012) and RR (*p* = 0.015) than 150 ml/min (Supplementary Table 1). The results of subgroup comparison of different anticoagulation methods showed that the improvement of HR (*p* = 0.009) and RR (*p* = 0.019) was heparin (36.54%, 41.38%, respectively), no anticoagulation (23.79%, 30.73%, respectively), and citrate (11.74%, 11.88%, respectively) in order (Supplementary Table 1).

#### Regression analysis

We conducted a logistic regression analysis for patient age, pre-treatment SOFA score, percentage SOFA score decline, APACHE II score, pre-treatment creatinine level, total CRRT duration, oXiris duration, time from ICU to oXiris initiation, number of oXiris-filters use, percentage MAP increase, percentage HR decline, percentage RR decline, percentage PCT decline, and percentage IL-6 decline. The results showed that pre-treatment SOFA score (*p* = 0.034), percentage decrease in SOFA score (*p* = 0.004), and age (*p* = 0.011) were independently associated with ICU mortality rate. For every 1-unit increase in pre-treatment SOFA score, the risk of death increased by 27%; for every 1 year of age increase, and 8.6%, the risk of death increased by 8.6%; for every 1 increase in percentage decrease in SOFA score, a 6.6% reduction in risk of death.

### Adverse events

**Figure 5:**
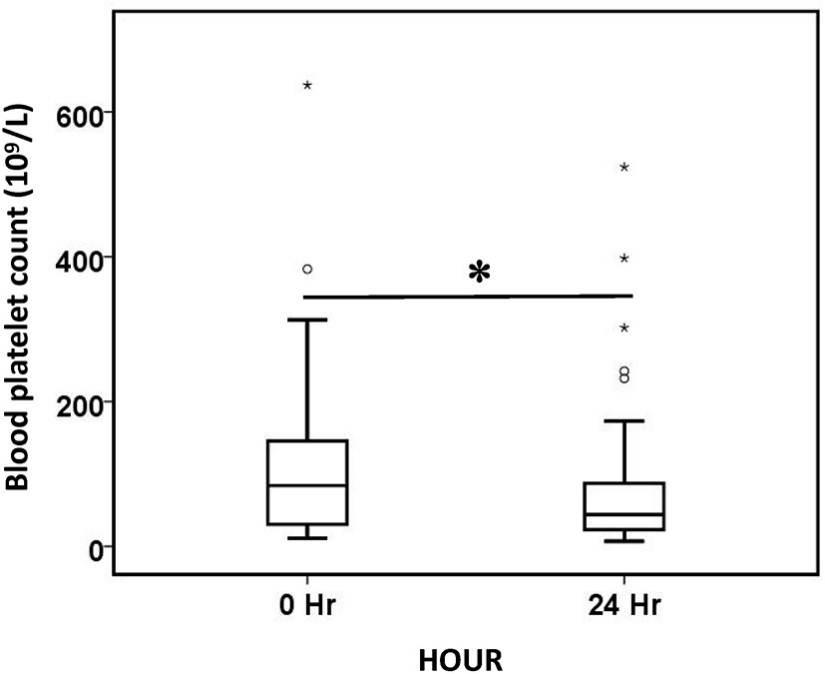
Comparison of blood platelet count before (0 h) and after (24 h) oXiris-CHFA treatment. *P < 0.001. The symbol * stands for outlier.

Blood platelet count decreased by 47.3% after 24 h of treatment (Figure 5). However, the decrease in platelets did not differ significantly between the different anticoagulation groups (*p* = 0.054), and it did not differ significantly among the different CRRT modality (*p* = 0.905). The majority of patients (83.3%) tolerated oXiris well; 15 patients experienced adverse events during treatment, with the most common being coagulation-related adverse events. 8 patients had high transmembrane pressure alarm in the treatment, 3 patients developed clotting deaeration chamber, 2 patients had hypotension during treatment, and 2 patients had abnormal pressure alarm at the arterial or venous end of the catheter.

## Discussion

The Third International Consensus Definitions for Sepsis and Septic Shock (Sepsis-3) defined sepsis as a life-threatening organ dysfunction caused by a dysregulated host response to infection. This organ dysfunction can be identified as an acute change in the total SOFA score of 2 after infection. Septic shock is classified as a subtype of sepsis, defined as the need for a vasopressor to maintain MAP ≥65 mmHg despite adequate volume resuscitation, with serum lactate levels >2 mmol/L (18 mg/dL) (1).

According to a recent global survey, sepsis is a common disease worldwide. In 2017, 48.9 million cases of sepsis were reported worldwide, resulting in 11 million deaths, or 19.7% of all global deaths (13). Hospital mortality rate in patients with sepsis ranges from 15 to 30%, and the 1-year mortality rate is 35% (14–16). Septic shock has a higher risk of mortality, with hospital and 1-year mortality rates of 39–56 and 60%, respectively (15–17). Sepsis is not only an important public health problem (18, 19), it also presents a significant global economic burden (20).

Patients with sepsis undergo immune hyperactivation and experience a cytokine storm, which leads to multiple organ failure (3). Cytokine storm is a comprehensive term for generalized immune dysregulation characterized by systemic symptoms and systemic inflammation and multi-organ dysfunction (21). The severity of the cytokine storm is associated with patient prognosis in septic shock (22).

Measures to treat sepsis include antimicrobial application and infectious source control, optimization of hemodynamics (using fluid and vasoactive drugs), blood purification therapy, and immunomodulatory/targeted therapy. Different treatments may be required for each stage of sepsis (23), and timely intervention in the early stage of the cytokine storm has the potential to improve the prognosis of patients with sepsis. The treatment targeting inflammatory mediators has become a new target in the treatment of sepsis, and the removal of systemic inflammatory mediators can be accomplished by blood purification therapy (3). Alleviating or eliminating endotoxin and cytokine storms in the body can be accomplished in various ways, including blood purification, which helps alleviate sepsis, improve patient hemodynamic status, and perhaps improve patient outcomes (3, 24–26).

Many different attempts have been made in the field of blood purification. For example, increasing the therapeutic dose, adjusting the interception molecular weight size, and using plasma exchange (27–31). A reasonable reason for the use of adsorption therapy in sepsis is the response to restoring balanced pro-inflammatory and anti-inflammatory mediators (32). Adsorption therapy is widely used in the treatment of sepsis and includes the use of cytokine adsorption columns (CytoSorb^®^) and polymyxin B adsorption columns (Toraymyxin^®^) (3, 32–35).

oXiris is a representative film material with high-adsorption film technology comprising three different layers (7). Its structure include AN69 copolymer hydrogel structures (to adsorb cytokines), multiple layers of polyethyleneimine (to adsorb endotoxins), and heparin grafting (to reduce local thrombogenicity). This unique design allows for a combination of four characteristics in one device: renal support, cytokine removal, endotoxin removal, and local anticoagulant therapy (7). It not only has high adsorption efficiency for cytokines and circulating endotoxin, but also has good blood compatibility, and can perform CHFA for patients without plasma separation. Recent studies have shown that the use of oXiris in patients with sepsis or septic shock can effectively reduce lactate levels, reduce concentrations of endotoxin and cytokines, optimize hemodynamics, reduce SOFA scores, and improve clinical outcomes (8, 9, 11, 36, 37). European Experience recommends oXiris in septic patients with unstable hemodynamic status, with or without AKI (38). The Asia Pacifica Experience also recommends that patients with sepsis or septic shock should be treated based on their hemodynamic indicators, microcirculation, and organ function, rather than AKI (39). Therefore, we believe that oXiris-based clinical research should be focused on patients' hemodynamic indicators and perfusion index and whether adverse reactions occurred in order to analyze the clinical outcome in terms of survival with oXiris treatment; this could provide a meaningful basis for the clinical treatment of sepsis.

In this study, a total of 90 patients with sepsis (median APACHE II: 24) were included; this cohort included patients with both ESRD and AKI. The initial SOFA score (median SOFA: 14) suggested that most patients had failure of more than two organs.

After 24 h of oXiris-CHFA treatment, we observed a 9.1% increase in MAP, 61.53% decreased in NE dose, 61.53% decreased in HR, 21.74% decreased in RR, and 37.86% decreased in lactate, suggesting that this treatment may improve hemodynamics and microcirculation perfusion in patients, showing the effectiveness of treatment intuitively in clinical situations. Among the parameters examined, the changes in lactate and improvement of hemodynamic indicators also appeared in parallel, consistent with the pathophysiological mechanism seen in sepsis patients. After oXiris-CHFA treatment combined with standard flow sepsis fluid resuscitation treatment, most patients seemed to obtain satisfactory hemodynamic status in a relatively short time (24 h), and their tissue perfusion quickly improved along with correction of internal environmental disorders such as hyperlactemia and subsequent acidosis caused by tissue hypoxia.

In terms of organ function maintenance, oXiris-CHFA treatment resulted in a decrease in SOFA score by 37.86%, and SOFA score decreased from higher high level of 14 (10.00–17.00) to lower level of 11.00 (9.00–15), indicating that this treatment can play a positive role in organ function maintenance in septic patients. A SOFA score change over 2 points is a reliable predictor of in-hospital mortality within the ICU (40). This change was very significant in the first 24 h of the first use of oXiris, suggesting that the use of this treatment as soon as possible may shorten the time of organ dysfunction, reduce the use of supportive care such as ventilators, vasoactive drugs, and blood products, and further shorten the organ support time of patients and reduce medical costs. The subsequent subgroup analysis also showed that although the difference in SOFA scores between the pre-treatment survival and non-survival groups was not significant, the SOFA scores in the survival group after oXiris-CHFA treatment were significantly lower than those in the non-survival group, indicating that functional status of the organs and improved prognosis were better in the former group of patients.

In terms of infection index, PCT showed a significant decrease after treatment (48.79%), while the IL-6 decrease was more obvious (81.80%). AS a widely used biomarker of sepsis, PCT is a precursor of calcitonin with extreme low level in general. However, almost all tissues and organs secrete PCT in pathological conditions and its generation is regulated by bacterial toxins and cytokines (41). It is used to guide the diagnosis and antibiotic treatment of sepsis (42–44), and it is also used as an indicator to evaluate the severity of sepsis (45). Its elevated concentration and non-clearance are closely related to the all-cause mortality of sepsis (46). IL-6, a well-known proinflammatory factor in cytokine storm, is a product of T cells that stimulates B cells and enhances antibody production. Together with IL-1 and the inflammatory mediator TNF, it is the main regulator of inflammation and one of the few true pleomorphic cytokines (47, 48). A decrease in IL-6 predicts the success rate of antibiotic therapy for sepsis in nonsurgical patients (49). Moreover, the dynamic change of IL-6 is closely related to the individual patient mortality rate (22). The IL-6 and PCT tests have similar diagnostic values in distinguishing sepsis from non-infectious systemic inflammatory response syndrome (50). Declines in the above two indicators show that after oXiris-CHFA treatment, the cytokine storm level of inflammatory cytokines in the body decreased significantly, and the systemic inflammatory response state was improved relative to that before treatment. The application of oXiris treatment in the early stage can help correct the high inflammation state of patients as soon as possible (within 24 h), reduce the resulting organ function damage, and reverse hemodynamic instability, thus improving the prognosis of patients.

In the present study, the ICU mortality, 30-day mortality, and hospital mortality rates were 34.4, 44.4, and 35.6%, respectively, consistent with previously reported mortality rates from sepsis and septic shock (14–17). Given that more-critical patients, often with more severe inflammatory responses, were included, the physician in charge preferred to use oXiris-CHFA; thus, all included patients had severe sepsis. This is confirmed by the higher SOFA score (14) and APACHE II score (24) at baseline, and we further discuss the factors influencing mortality rate in the subsequent subgroup analysis results. Due to the different timing of inclusion, although 77 (85.56%) had 2 mmol/L lactate before oXiris-CHFA and 84 patients (87.00%) needed NE to maintain blood pressure, the actual number of patients with septic shock may not have had adequate fluid resuscitation.

The median time from ICU admission to initiation of oXiris treatment was 18 h, somewhat earlier than the 21–46 h in several other studies (11, 37, 51). The ICU mortality rate was also lower than previously reported rates (37). A retrospective study showed that patients who were started on oXiris treatment within 3 h of adequate resuscitation had reduced vasopressor use, decreased SOFA scores, and increased MAP compared with those who were started on oXiris after 3 h of adequate resuscitation (52). However, in this study, there was no significant difference in ICU mortality rate between patients initiated with oXiris-CHFA within 24 h versus after 24 h after admission (*p* = 0.921). A detailed review of the medical history showed that some patients were admitted to the ICU with a first diagnosis of severe pneumonia or hemorrhagic shock, liver and kidney failure, or cardiac and respiratory arrest rather than sepsis. In the course of ICU hospitalization, sepsis and septic shock occurred. That is, there was no indication for initiating oXiris-CHFA therapy when the patient first entered the ICU. Moreover, there are many factors affecting patient mortality, and inflammatory adsorption treatment is only part of the comprehensive treatment.

By comparing the two groups of patients in this study with prescribed therapeutic doses ≥30 mL/kg/h and <30 mL/kg/h, it appeared that no prescribed therapeutic dose was directly associated with ICU mortality rate. We found that the prescribed therapeutic doses of the included patients were basically within the appropriate range of the prescribed therapeutic doses of 25–30 mL/kg/h (actual achieved therapeutic dose 20–25 mL/kg/h) recommended by the Kidney Disease Improving Global Outcomes (KDIGO) guidelines (12). oXiris-CHFA–mediated improvement in endotoxin and cytokine storms in septic patients occurred mainly through the adsorption of the membrane (7). This effect is mainly related to the membrane area and filter use time rather than therapeutic dose, which may also be a prime reason for the above result.

Sepsis is a clinical syndrome with great heterogeneity rather than a class of diseases with relatively consistent etiology and pathophysiology (3). Numerous factors affect the prognosis of sepsis patients and include multi-organ system support treatment, volume management, etiology treatment, and rehabilitation treatment, CRRT does not independently influence patient treatment.

The therapeutic effect of oXiris-CHFA varies based on the primary site of infection in sepsis. The top three primary infection sites of inflammatory indicators (PCT and IL-6 decline) in this study were urinary tract, abdominal cavity, and skin and soft tissue, indicating that oXiris-CHFA treatment may be more clinically effective for infections originating from these regions. This is also consistent with previous reports that treatment with oXiris significantly improved hemodynamics and inflammation in patients with sepsis/septic shock due to abdominal, urinary, and skin soft tissue infections (8, 11, 53). Binary logistic regression analysis showed that both initial status and severity of decline in SOFA score were independently associated with ICU mortality. This shows that the more severe the organ failure before treatment, the higher the risk of death; the better the organ function recovery after treatment, the lower the risk of death. This finding is consistent with previous studies (18, 54).

From our results, oXiris-CHFA treatment initiated in the early stage of sepsis (AKI stage 1) may reduce the levels of proinflammatory factors such as IL-6 more significantly than in the later stage (AKI stage 2–3). It can also be understood that the main purpose of oXiris-CHFA is not to replace the kidney, but to clear the early inflammatory storm, which is also consistent with many previous literatures (3, 38, 39). As for the effect of blood flow rate and anticoagulation on the improvement of vital signs, we all know that blood flow rate is closely related to the anticoagulation method, and also related to the basal state of the patient. In the case of heparin anticoagulation or no anticoagulation, the blood flow rate of CRRT is usually 200 ml/min, while the blood flow rate of citrate anticoagulation is usually <150 ml/min to ensure the anticoagulation effect. We believe that in CRRT patients with heparin and without anticoagulation, a higher flow rate can be used to correct the possible volume overload in these patients more quickly, resulting in more significant improvements in HR and RR.

Platelet counts decreased after oXiris-CHFA treatment, but this difference was not significant in subgroup analyses comparing anticoagulation (*p* = 0.054) and treatment modalities (*p* = 0.905). The reasons may be as follows. First, cardiopulmonary bypass lines activate blood coagulation. Although heparin has been pre-grafted in the oXiris filter, other parts of the extracorporeal line do not have anticoagulation efficacy, so anticoagulation is still activated, resulting in the consumption of coagulation substances and consequent decrease of platelet count. The results of this study suggest that thrombocytopenia is not caused by inadequate anticoagulation or improper mode setting. Second, diseases with active bleeding in the primary site or severe infection cause platelet decline. The above two reasons have been similarly reported in previous studies of case reports (53). Further studies need to explore whether oXiris will directly leadly to a decrease in platelet count. Other adverse events were mainly thrombosis-related adverse events, but no bleeding-related adverse events were seen. Studies have also shown that fixed heparin has no significant systemic anticoagulant or adverse bleeding events (55). This also suggests that we should regulate anticoagulation according to the condition of oXiris treatment to reduce activation of the coagulation system and the consumption of coagulation factors and platelets.

This study included 90 patients with different causes of sepsis and septic shock with improvement in hemodynamic parameters, lactate levels, and organ function after oXiris-CHFA treatment; recorded changes in infection and inflammatory indicators and platelet changes; and finally recorded patient outcome. The present study has some limitations. First, this is a descriptive small case series, lacking a control group with a heterogeneous group of patients (i.e., infectious source, duration of antibiotic administration, resuscitation regimen) and multiple concomitant interventions (e.g., CRRT, antibiotics, ECMO, hemoperfusion, and vasopressors). This is probably the main limitation of this article. There are three reasons for the absence of control in this study: (1) All sepsis patients in our hospital during the same period (from November 2020 to now) have been treated with oXiris. (2) If historical control is selected, given the rapid update of sepsis guidelines in recent years, especially in fluid resuscitation and hemodynamic management, which have a great impact on the results of this study, the control group can provide limited reference. (3) This study focuses on the changes in hemodynamics, perfusion level and organ function of patients before and after oXiris use, and whether oXiris has different effects on patients with different primary infections. Second, due to the retrospective study design, PCT or IL-6 results were missing in some cases. Third, some patients eventually abandoned treatment for discharge owing to non-medical factors, which may have had an impact on the final outcome. Finally, whether improvements in hemodynamic and metabolic parameters might be achieved only by infection control and CRRT itself, and not necessarily by oXiris-CHFA treatment. oXiris-CHFA treatment can be used as an adjuvant treatment for sepsis patients, but further randomized controlled trials with a larger sample size are needed.

## Conclusions

In patients with sepsis or septic shock, oXiris-CHFA treatment was associated with a significant improvement in hemodynamic measures, significantly decreased vasoactive drug dosage, reduced lactate level and infection measures, and decreased SOFA score after treatment. The SOFA score was an independent risk factor for ICU mortality. However, improvement of SOFA score after oXiris-CHFA treatment was scarcely reported and we provided convincing evidence in the present study. In terms of the primary site of infection, patients with skin and soft tissue, urinary tract, and abdominal cavity infections benefitted the most from treatment with oXiris-CHFA. The results of this study show that the efficacy and safety of oXiris-CHFA treatment are relatively high. It's worth noting that we observed no evidence that a therapeutic dose of ≥30 mL/kg/h improves survival rate of patient, and the decrease in platelet count may be multifactorial.

## Data availability statement

The raw data supporting the conclusions of this article will be made available by the authors, without undue reservation.

## Ethics statement

The studies involving human participants were reviewed and approved by the Institutional Ethics Committee of the Second Xiangya Hospital of Central South University. Written informed consent for participation was not required for this study in accordance with the national legislation and the institutional requirements.

## Author contributions

YZ and JL helped in conceptualization, writing of the original draft, data curation, formal analysis, methodology, organization of results, assisted with conceptualization, fund acquisition, supervision, and writing-review and editing. YP, DZ, and XX performed data collection. YZ, CW, and LO analyzed the data.

## Funding

This work was supported by the Scientific Research Project of the Natural Science Foundation of Changsha (grant number: kq2202413).

## Conflict of interest

The authors declare that the research was conducted in the absence of any commercial or financial relationships that could be construed as a potential conflict of interest.

## Publisher's note

All claims expressed in this article are solely those of the authors and do not necessarily represent those of their affiliated organizations, or those of the publisher, the editors and the reviewers. Any product that may be evaluated in this article, or claim that may be made by its manufacturer, is not guaranteed or endorsed by the publisher.

## References

[1] Singer M Deutschman CS Seymour CW Shankar-Hari M Annane D Bauer M. The third international consensus definitions for sepsis and septic shock (Sepsis-3). JAMA. (2016) 315:801–10. 10.1001/jama.2016.0287 26903338 PMC4968574

[2] Leligdowicz A Matthay MA. Heterogeneity in sepsis: new biological evidence with clinical applications. Crit Care. (2019) 23:80. 10.1186/s13054-019-2372-2 30850013 PMC6408778

[3] Zhang Y-Y Ning B-T. Signaling pathways and intervention therapies in sepsis. Signal Transduct Target Ther. (2021) 6:407. 10.1038/s41392-021-00816-9 34824200 PMC8613465

[4] Hellman T Uusalo P Järvisalo MJ. Renal replacement techniques in septic shock. Int J Mol Sci. (2021) 22:10238. 10.3390/ijms221910238 34638575 PMC8508758

[5] Karkar A Ronco C. Prescription of crrt: a pathway to optimize therapy. Ann Intensive Care. (2020) 10:32. 10.1186/s13613-020-0648-y 32144519 PMC7060300

[6] Yang Q Li Y Tuohuti P Qin Z Zhang Z Zhao W. Advances in the development of biomaterials for endotoxin adsorption in sepsis. Front Bioeng Biotechnol. (2021) 9:699418. 10.3389/fbioe.2021.699418 34395405 PMC8361450

[7] Monard C Rimmelé T Ronco C. Extracorporeal blood purification therapies for sepsis. Blood Purif . (2019) 47 (Suppl. 3):1–14. 10.1159/000499520 30974444

[8] Broman ME Hansson F Vincent J-L Bodelsson M. Endotoxin and cytokine reducing properties of the oxiris membrane in patients with septic shock: a randomized crossover double-blind study. PLoS ONE. (2019) 14:e0220444. 10.1371/journal.pone.0220444 31369593 PMC6675097

[9] Zhai Y Pan J Zhang C. The application value of oxiris-endotoxin adsorption in sepsis. Am J Transl Res. (2021) 13:3839–44. 34017574 PMC8129347

[10] Li Y Ji X Jing D Huang Z Duan M. Successful treatment of gastrointestinal infection-induced septic shock using the oxiris hemofilter: a case report. World J Clin Cases. (2021) 9:8157–63. 10.12998/wjcc.v9.i27.8157 34621875 PMC8462223

[11] Schwindenhammer V Girardot T Chaulier K Grégoire A Monard C Huriaux L. Oxiris^®^ use in septic shock: experience of two french centres. Blood Purif . (2019) 47 (Suppl. 3):1–7. 10.1159/000499510 30982028

[12] Kellum JA Lameire N Aspelin P Barsoum RS Burdmann EA Goldstein SL. Kidney disease: Improving global outcomes (KDIGO) acute kidney injury work group. KDIGO clinical practice guideline for acute kidney injury. Kidney Int Suppl. (2012) 2:1–138. 10.1038/kisup.2012.1

[13] Rudd KE Johnson SC Agesa KM Shackelford KA Tsoi D Kievlan DR. Global, regional, and national sepsis incidence and mortality, 1990-2017: analysis for the global burden of disease study. Lancet. (2020) 395:200–11. 10.1016/S0140-6736(19)32989-7 31954465 PMC6970225

[14] Rhee C Dantes R Epstein L Murphy DJ Seymour CW Iwashyna TJ. Incidence and trends of sepsis in US hospitals using clinical vs claims data, 2009-2014. JAMA. (2017) 318:1241–9. 10.1001/jama.2017.13836 28903154 PMC5710396

[15] Shankar-Hari M Harrison DA Rubenfeld GD Rowan K. Epidemiology of sepsis and septic shock in critical care units: comparison between sepsis-2 and sepsis-3 populations using a national critical care database. Br J Anaesth. (2017) 119:626–36. 10.1093/bja/aex234 29121281

[16] Laupland KB Zygun DA Doig CJ Bagshaw SM Svenson LW Fick GH. One-year mortality of bloodstream infection-associated sepsis and septic shock among patients presenting to a regional critical care system. Intens Care Med. (2005) 31:213–9. 10.1007/s00134-004-2544-6 15666140

[17] Vincent J-L Jones G David S Olariu E Cadwell KK. Frequency and mortality of septic shock in Europe and North America: a systematic review and meta-analysis. Crit Care. (2019) 23:196. 10.1186/s13054-019-2478-6 31151462 PMC6545004

[18] Fleischmann C Scherag A Adhikari NKJ Hartog CS Tsaganos T Schlattmann P. Assessment of global incidence and mortality of hospital-treated sepsis. Current estimates and limitations. Am J Respir Crit Care Med. (2016) 193:259–72. 10.1164/rccm.201504-0781OC 26414292

[19] Markwart R Saito H Harder T Tomczyk S Cassini A Fleischmann-Struzek C. Epidemiology and burden of sepsis acquired in hospitals and intensive care units: a systematic review and meta-analysis. Intens Care Med. (2020) 46:1536–51. 10.1007/s00134-020-06106-2 32591853 PMC7381455

[20] Tiru B DiNino EK Orenstein A Mailloux PT Pesaturo A Gupta A. The economic and humanistic burden of severe sepsis. Pharmacoeconomics. (2015) 33:925–37. 10.1007/s40273-015-0282-y 25935211

[21] Fajgenbaum DC June CH. Cytokine Storm. N Engl J Med. (2020) 383:2255–73. 10.1056/NEJMra2026131 33264547 PMC7727315

[22] Abasiyanik MF Wolfe K Van Phan H Lin J Laxman B White SR. Ultrasensitive digital quantification of cytokines and bacteria predicts septic shock outcomes. Nat Commun. (2020) 11:2607. 10.1038/s41467-020-16124-9 32451375 PMC7248118

[23] Wang M. Sepsis gene signatures over time and space. Nat Rev Nephrol. (2021) 17:221. 10.1038/s41581-021-00401-x 33531662

[24] Wendel Garcia PD Hilty MP Held U Kleinert E-M Maggiorini M. Cytokine adsorption in severe, refractory septic shock. Intens Care Med. (2021) 47:1334–6. 10.1007/s00134-021-06512-0 34471938 PMC8409473

[25] Davies B Cohen J. Endotoxin removal devices for the treatment of sepsis and septic shock. Lancet Infect Dis. (2011) 11:65–71. 10.1016/S1473-3099(10)70220-6 21183148

[26] Kang JH Super M Yung CW Cooper RM Domansky K Graveline AR. An extracorporeal blood-cleansing device for sepsis therapy. Nat Med. (2014) 20:1211–6. 10.1038/nm.3640 25216635

[27] Borthwick EM Hill CJ Rabindranath KS Maxwell AP McAuley DF Blackwood B. High-volume haemofiltration for sepsis in adults. Cochrane Database Syst Rev. (2017) 1:CD008075. 10.1002/14651858.CD008075.pub3 28141912 PMC6464723

[28] Garbero E Livigni S Ferrari F Finazzi S Langer M Malacarne P. High dose coupled plasma filtration and adsorption in septic shock patients. Results of the compact-2: a multicentre, adaptive, randomised clinical trial. Intens Care Med. (2021) 47:1303–11. 10.1007/s00134-021-06501-3 34601619

[29] Joannes-Boyau O Honoré PM Perez P Bagshaw SM Grand H Canivet J-L. High-volume versus standard-volume haemofiltration for septic shock patients with acute kidney injury (Ivoire Study): a multicentre randomized controlled trial. Intens Care Med. (2013) 39:1535–46. 10.1007/s00134-013-2967-z 23740278

[30] Villa G Chelazzi C Morettini E Zamidei L Valente S Caldini AL. Organ dysfunction during continuous veno-venous high cut-off hemodialysis in patients with septic acute kidney injury: a prospective observational study. PLoS ONE. (2017) 12:e0172039. 10.1371/journal.pone.0172039 28207795 PMC5313216

[31] David S Bode C Putensen C Welte T Stahl K. Adjuvant therapeutic plasma exchange in septic shock. Intens Care Med. (2021) 47:352–4. 10.1007/s00134-020-06339-1 33471132 PMC7816555

[32] Ankawi G Xie Y Yang B Xie Y Xie P Ronco C. What have we learned about the use of cytosorb adsorption columns? Blood Purif. (2019) 48:196–202. 10.1159/000500013 31039564

[33] Dellinger RP Bagshaw SM Antonelli M Foster DM Klein DJ Marshall JC. Effect of Targeted Polymyxin B Hemoperfusion on 28-day mortality in patients with septic shock and elevated endotoxin level: the euphrates randomized clinical trial. JAMA. (2018) 320:1455–63. 10.1001/jama.2018.14618 30304428 PMC6233793

[34] Shoji H Opal SM. Therapeutic rationale for endotoxin removal with Polymyxin B immobilized fiber column (Pmx) for septic shock. Int J Mol Sci. (2021) 22:2228. 10.3390/ijms22042228 33672437 PMC7926968

[35] Pickkers P Russell JA. Treatment with a Polymyxin B filter to capture endotoxin in sepsis patients: is there a signal for therapeutic efficacy? Intens Care Med. (2019) 45:282–3. 10.1007/s00134-018-5481-5 30511248

[36] Turani F Barchetta R Falco M Busatti S Weltert L. Continuous renal replacement therapy with the adsorbing filter oxiris in septic patients: a case series. Blood Purif . (2019) 47 (Suppl. 3):1–5. 10.1159/000499589 30982024

[37] Zang S Chen Q Zhang Y Xu L Chen J. Comparison of the clinical effectiveness of An69-oxiris versus An69-st filter in septic patients: a single-centre study. Blood Purif. (2022) 51:617–29. 10.1159/000519166 34610595

[38] Pickkers P Vassiliou T Liguts V Prato F Tissieres P Kloesel S. Sepsis management with a blood purification membrane: european experience. Blood Purif . (2019) 47 (Suppl. 3):1–9. 10.1159/000499355 30982031

[39] Zhang L Cove M Nguyen BG Lumlertgul N Ganesh K Chan A. Adsorptive hemofiltration for sepsis management: expert recommendations based on the asia pacific experience. Chin Med J (Engl). (2021) 134:2258–60. 10.1097/CM9.0000000000001671 34402478 PMC8478384

[40] Raith EP Udy AA Bailey M McGloughlin S MacIsaac C Bellomo R. Prognostic accuracy of the sofa score, sirs criteria, and qsofa score for in-hospital mortality among adults with suspected infection admitted to the intensive care unit. JAMA. (2017) 317:290–300. 10.1001/jama.2016.20328 28114553

[41] Assicot M Gendrel D Carsin H Raymond J Guilbaud J Bohuon C. High serum procalcitonin concentrations in patients with sepsis and infection. Lancet. (1993) 341:515–8. 10.1016/0140-6736(93)90277-N 8094770 PMC7141580

[42] Kyriazopoulou E Liaskou-Antoniou L Adamis G Panagaki A Melachroinopoulos N Drakou E. Procalcitonin to reduce long-term infection-associated adverse events in sepsis. A randomized trial. Am J Respir Crit Care Med. (2021) 203:202–10. 10.1164/rccm.202004-1201OC 32757963 PMC7874409

[43] Shehabi Y Sterba M Garrett PM Rachakonda KS Stephens D Harrigan P. Procalcitonin algorithm in critically ill adults with undifferentiated infection or suspected sepsis. A randomized controlled trial. Am J Respir Crit Care Med. (2014) 190:1102–10. 10.1164/rccm.201408-1483OC 25295709

[44] Vouloumanou EK Plessa E Karageorgopoulos DE Mantadakis E Falagas ME. Serum procalcitonin as a diagnostic marker for neonatal sepsis: a systematic review and meta-analysis. Intens Care Med. (2011) 37:747–62. 10.1007/s00134-011-2174-8 21380522

[45] Rey C Los Arcos M Concha A Medina A Prieto S Martinez P. Procalcitonin and C-reactive protein as markers of systemic inflammatory response syndrome severity in critically ill children. Intens Care Med. (2007) 33:477–84. 10.1007/s00134-006-0509-7 17260130

[46] Liu D Su L Han G Yan P Xie L. Prognostic value of procalcitonin in adult patients with sepsis: a systematic review and meta-analysis. PLoS ONE. (2015) 10:e0129450. 10.1371/journal.pone.0129450 26076027 PMC4468164

[47] Hirano T Yasukawa K Harada H Taga T Watanabe Y Matsuda T. Complementary DNA for a novel human interleukin (Bsf-2) that induces B lymphocytes to produce immunoglobulin. Nature. (1986) 324:73–6. 10.1038/324073a0 3491322

[48] Copaescu A Smibert O Gibson A Phillips EJ Trubiano JA. The role of Il-6 and other mediators in the cytokine storm associated with SARS-CoV-2 infection. J Allergy Clin Immunol. (2020) 146:518:534. 10.1016/j.jaci.2020.07.001 32896310 PMC7471766

[49] Weidhase L Wellhöfer D Schulze G Kaiser T Drogies T Wurst U. Is Interleukin-6 a better predictor of successful antibiotic therapy than procalcitonin and C-reactive protein? A single center study in critically Ill adults. BMC Infect Dis. (2019) 19:150. 10.1186/s12879-019-3800-2 30760225 PMC6375140

[50] Ma L Zhang H Yin Y-L Guo W-Z Ma Y-Q Wang Y-B. Role of interleukin-6 to differentiate sepsis from non-infectious systemic inflammatory response syndrome. Cytokine. (2016) 88:126–35. 10.1016/j.cyto.2016.08.033 27599258

[51] Shum HP Chan KC Kwan MC Yan WW. Application of endotoxin and cytokine adsorption haemofilter in septic acute kidney injury due to gram-negative bacterial infection. Hong Kong Med J. (2013) 19:491–7. 10.12809/hkmj133910 23650198

[52] Govil D Gupta S Srinivasan S Patel SJ Jagadeesh KN Shafi M. 054 cytokine adsorption in sepsis: correct timing can predict the favorable outcome. Kidney Int Rep. (2017) 2:S29. 10.1016/j.ekir.2017.06.096

[53] Zhang L Yan Tang GK Liu S Cai J Chan WM Yang Y. Hemofilter with adsorptive capacities: case report series. Blood Purif . (2019) 47 (Suppl. 3):1–6. 10.1159/000499357 30982026

[54] Pölkki A Pekkarinen PT Takala J Selander T Reinikainen M. Association of sequential organ failure assessment (Sofa) components with mortality. Acta Anaesthesiol Scand. (2022) 66:732–41. 10.21203/rs.3.rs-556787/v1 35353902 PMC9322581

[55] Wong ET-Y Ong V Remani D Wong W-K Haroon S Lau T. Filter life and safety of heparin-grafted membrane for continuous renal replacement therapy - a randomized controlled trial. Semin Dial. (2021) 34:300–8. 10.1111/sdi.12951 33556204

