# Supplementary material for: Application of oXiris-continuous hemofiltration adsorption in patients with sepsis and septic shock: A single-centre experience in China
Source: Front Public Health. 2022 Sep 29;10:1012998. doi: 10.3389/fpubh.2022.1012998 (PMC9557776; doi:10.3389/fpubh.2022.1012998)
Supplement: Supplementary file 1 [file Table_1.DOCX]

**Supplementary Table 1.** Subgroup analysis of oXiris-CHFA for treatment of sepsis

| Groups | Percentage reduction of (%) | | | | | | | Percentage increase of MAP(%) |
| --- | --- | --- | --- | --- | --- | --- | --- | --- |
|  | PCT | IL-6 | SOFA | lactate | HR | RR | NE dose |  |
| Renal function at CRRT initiation |  |  |  |  |  |  |  |  |
| AKI Stage 3 | 32.71 | 75.34 | 6.25 | 39.29 | 20.16 | 27.27 | 69.57 | 4.96 |
| AKI Stage 2 | 43.06 | 64.64 | 2.78 | 11.15 | 19.82 | 16.53 | 79.58 | 10.94 |
| AKI Stage 1 | 29.43 | 84.83 | 10.0 | 17.65 | 11.81 | 9.09 | 59.17 | 0 |
| ESRD | 39.0 | 14.28 | 7.14 | 7.14 | 25.0 | 35.71 | 40.83 | 13.64 |
| p^a^ value | 0.984 | 0.027 | 0.824 | 0.887 | 0.557 | 0.190 | 0.073 | 0.054 |
| Site of infection |  |  |  |  |  |  |  |  |
| Pulmonary | 3.57 | 23.64 | 0 | 16.28 | 28.13 | 33.33 | 50.0 | 10.67 |
| Abdominal | 43.64 | 79.29 | 8.71 | 23.40 | 8.0 | 11.11 | 65.15 | 2.5 |
| Skin and soft tissue | 61.4 | 80.42 | 2.78 | -17.64 | 20.97 | 32.54 | 77.64 | 6.93 |
| Bacteremia | 7.35 | 0 | 8.11 | 30.77 | 22.96 | 31.82 | 25.0 | 12.99 |
| Urinary | 57.2 | 93.8 | 25.71 | 24.44 | 11.10 | 23.64 | 78.57 | 20.89 |
| p^a^ value | 0.035 | 0.001 | 0.424 | 0.729 | 0.172 | 0.162 | 0.365 | 0.137 |
| Culture |  |  |  |  |  |  |  |  |
| Gram negative | 12.70 | 80.00 | 6..67 | 16.67 | 22.55 | 25.93 | 63.63 | 6.41 |
| Gram positive | 37.62 | 47.86 | 5.72 | 16.96 | 17.11 | 16.53 | 69.57 | 6.13 |
| Fungus | 54.69 | 47.46 | 0 | 28.30 | 20.16 | 23.81 | 25.00 | 12.99 |
| Not identified | 43.06 | 55.99 | 21.43 | 4.35 | 13.79 | 13.64 | 80.46 | 10.04 |
| p^a^ value | 0.471 | 0.245 | 0.172 | 0.796 | 0.886 | 0.834 | 0.115 | 0.918 |
| CRRT modality |  |  |  |  |  |  |  |  |
| CVVH | 39.01 | 74.85 | 7.14 | 17.65 | 21.64 | 30.43 | 60.95 | 9.59 |
| CVVH+HP | 58.1 | 82.16 | 13.84 | 15.30 | 23.27 | 15.66 | 75.0 | 1.77 |
| CVVHDF | 16.28 | 76.67 | 5.72 | 25.78 | 12.80 | 9.94 | 70.0 | 10.21 |
| p^a^ value | 0.874 | 0.885 | 0.872 | 0.978 | 0.510 | 0.145 | 0.515 | 0.882 |
| Blood flow rate |  |  |  |  |  |  |  |  |
| 150 mL/min | 43.06 | 80.99 | 7.74 | 18.50 | 11.74 | 11.88 | 54.17 | 5.31 |
| 200 mL/min | 31.07 | 51.13 | 6.67 | 23.30 | 25.71 | 31.82 | 73.33 | 11.43 |
| p^a^ value | 0.761 | 0.176 | 0.722 | 0.440 | 0.012 | 0.015 | 0.183 | 0.192 |
| Circuit anticoagulation |  |  |  |  |  |  |  |  |
| Citrate | 43.06 | 80.99 | 7.74 | 18.50 | 11.74 | 11.88 | 54.17 | 5.31 |
| Heparin | -89.45 | 55.46 | 9.09 | -11.76 | 36.54 | 41.38 | 20.00 | 20.00 |
| None | 41.11 | 51.12 | 6.27 | 31.49 | 23.79 | 30.73 | 73.33 | 11.41 |
| p^a^ value | 0.097 | 0.350 | 0.937 | 0.315 | 0.009 | 0.019 | 0.250 | 0.402 |
| Prescription therapeutic dose |  |  |  |  |  |  |  |  |
| ≥30 mL/kg/h | 40.39 | 75.34 | 0 | 19.35 | 19.71 | 23.02 | 65.15 | 7.11 |
| ＜30 mL/kg/h | 16.29 | 69.52 | 9.55 | 19.35 | 22.55 | 20.00 | 66.67 | 10.45 |
| p^a^ value | 0.431 | 0.844 | 0.106 | 0.771 | 0.996 | 0.419 | 0.695 | 0.706 |

AKI: acute kidney injury; CRRT: continuous renal replacement therapy; ESRD: end-stage renal disease; CRRT: continuous renal replacement therapy; CVVH: continuous venovenous hemofiltration; HP: hemoperfusion; CVVHDF: continuous venovenous hemodiafiltration; ICU: intensive care unit. oXiris-CHFA: continuous hemofiltration adsorption with oXiris; MAP: mean arterial pressure; HR: heart rate; RR: respiratory rate; SOFA: Sequential Organ Failure Assessment. Non-normally distributed measurement data were expressed as median [interquartile range].

^a^Independent Sample Kruskal -- Wallis test was used for comparison among subgroups.
